# Implementation of a Cancer Education Program in Rural Counties with the Lowest HPV Vaccination Rates and Health Rankings

**DOI:** 10.56808/2586-940x.1057

**Published:** 2023-09-14

**Authors:** Sarah Arezo, Shillpa Naavaal, Charlotte Garrett, Marcie S. Wright, Vanessa B. Sheppard, Michael A. Preston

**Affiliations:** a Virginia Commonwealth University, School of Medicine, Department of Health Behavior and Policy, 830 East Main Street, PO Box 980149, Richmond, VA 23298-0149, USA; b Virginia Commonwealth University, Massey Cancer Center, Health Equity & Disparities Research, 830 East Main Street, PO Box 980149, Richmond, VA 23298-0149, USA; c Virginia Commonwealth University, School of Medicine, Center on Health Disparities, 730 E. Broad St. 4th Floor, Richmond, VA 23219, USA; d Virginia Commonwealth University, School of Dentistry, Department of Dental Public Health and Policy, 520 North 12th Street, PO Box 980566, Richmond, VA 23298-0566, USA

**Keywords:** Human papillomavirus, Rural health, Cancer prevention, Schooling cancer program, Healthy lifestyle, Vaccination

## Abstract

**Background:**

Human papillomavirus (HPV) is the most common sexually transmitted infection (STI). To address STIs, one rural county public school district developed a series of Family Life Programs to educate pre-teens about pertinent health information. The Schooling Cancer Program (SCP) was developed in partnership with the local Cancer Research and Resource Center to raise awareness about cancer risk factors including HPV-related cancers and HPV prevention methods.

**Methods:**

We collected a post-evaluation survey from students who attended a SCP session at one of the targeted middle schools. The SCP educated students about topics focusing on healthy lifestyles. The survey asked students’ knowledge on the SCP topics, HPV knowledge, tobacco usage, and factors that reduced cancer development.

**Results:**

87% agreed that tobacco products are associated with cancer, and 81% did not agree that E-cigarettes are scientifically proven to be safer than cigarettes. Although we do not have pre-evaluation data about these students’ HPV knowledge, our evaluation survey shows that 80% of students correctly identified HPV as the most common STI, and 84% of students correctly identified the factors that decrease their risk of developing cancer.

**Conclusion:**

Through this initiative, students learned essential health concepts and HPV-related risk factors.

## 1. Introduction

Human papillomavirus (HPV) is a commonly transmitted viral infection [1,2]. HPV infection is linked with various types of cancers such as cervical cancer, anal cancer, oropharyngeal cancer, penile cancer, vaginal cancer, and valvular cancer [3,4]. An effective way of minimizing the risks of HPV infection is by getting the HPV vaccine, which is an important health promotion element in adolescents [3]. The Cancer Research and Resource Centers (CRRCs) at the NCI-Designated Massey Cancer Center function in rural areas located in Danville and Lawrenceville, Virginia that rank low in the Robert Wood Johnson Foundation Health Rankings. Nearly half of all cancers can be prevented through a healthy and active lifestyle. Based on the Positive Behavioral Interventions and Supports [5,6], the Schooling Cancer Program is a byproduct of the HPV/Cancer Awareness Task Force, which was created in 2014 to address cancer disparities in rural Virginia. The Schooling Cancer Program has been used to educate middle and high school-aged students about cancer prevention, assessing HPV knowledge, and how implementing a healthy lifestyle can decrease an individual’s risk of developing cancer.

The most common subtypes of HPV are subtype 16 and subtype 18 [2,3]. These subtypes are high-risk subtypes that can cause cancer development, which is why the HPV vaccine for school-aged children is crucial [3]. The Centers for Disease Control and Prevention (CDC) recommends that the HPV vaccine be given to children between ages 11 and 12. It can be given as early as age 9. CDC’s Advisory Committee on Immunization Practices also recommends vaccination for everyone through age 26 years if not adequately vaccinated when younger. HPV vaccination is given as a series of either two or three doses, depending on age at initial vaccination [2,3,7,8]. Research indicates that the decision to vaccinate a child is complex and many factors influence the decision, including cultural, social, and familial factors [3]. In many rural and underserved communities, adopting health promotion strategies, establishing communication, and forming rapport between caregivers and healthcare providers is crucial in helping an individual to make an informed vaccination decision and educating the younger population on the virus [3]. The uptake of HPV vaccinations in rural areas and among racial minorities in areas with high poverty is associated with factors such as vaccine acceptance, health care facilities in the area, education available in school districts, and access to HPV vaccines through the Vaccines for Children Program, which is a program that provides under-insured and uninsured children with the necessary vaccinations [9]. Findings suggest that urban areas and highly populated areas have a higher vaccination coverage percentage compared to rural and suburban areas [9].

Thanasas’s study and previously by Dell’s study assessed the knowledge of HPV among high school students showed that only a small percentage (25%) were estimated to have heard about HPV and vaccination [10,11]. Dell’s study showed that 87% of the students had not heard of HPV [11]. General knowledge about HPV and related cancers as well as cervical cancer screening was low among the adolescent population [10,11]. Programs that are geared towards HPV prevention, such as Schooling Cancer and healthcare clinics that provide HPV information to adolescents, will be useful in educating students about the risks of HPV [6,10,11]. Accurate sexual education knowledge is correlated to positive attitudes towards the HPV vaccine, which can ultimately lead to cancer prevention among many adolescents. HPV knowledge should be integrated into school-based Family Life courses to increase the acceptability of the HPV vaccine, as well as to provide students with the necessary information to maintain a healthy lifestyle [12].

### 1.1. Populations

The CRRCs outreach team went to Pittsylvania County, Virginia, to speak with students about HPV prevention and health. While speaking with the students, team members primarily focused on the topic of risky behaviors that begin at a young age and how they often have long-term effects on health. To increase the acceptance of HPV vaccinations among middle schoolers, effective school-based interventions focusing on HPV and its related cancers should be implemented into the school systems to raise awareness on HPV prevention [12]. Research has shown that students have strong fears and anxieties towards the HPV vaccine, causing hesitancy [13].

Pittsylvania County is geographically the largest county in the state of Virginia with a large rural population. This rural community has a strong enduring agricultural history, where tobacco was once the widely used cash crop. Every day in the United States, around 4000 young adults and adolescents begin smoking and form a nicotine addiction [14]. Cigarette smoking and tobacco usage are important factors that have been linked to increased risks of developing HPV-related cancers. Meanwhile, tobacco smoking and secondhand exposure to tobacco haven both been linked to increased chances of having genital HPV or high-risk HPV infection [15]. Considering how difficult it is to receive adequate health education in rural areas, rural regions and schools need additional assistance in receiving and disseminating health information, which is why the Schooling Cancer Program was created [16–18]. Research shows that parents who reside in rural communities are misinformed about “reproductive health issues [such as] HPV,” which can lead to vaccine hesitancy [3]. The need to address cancer prevention and HPV in rural areas is crucial. Cancer prevention education in rural areas is not prioritized, and the “complexities involved in educating parents must be addressed in the context of the local rural culture” as well as with community engagement [19]. To better help rural communities, we must understand cultural and parental perspectives to develop useful interventions and increase HPV vaccination rates in these rural communities [19].

### 1.2. Methodology

The Schooling Cancer Program was a 1-h program created to educate middle school students in Pittsylvania County about HPV, to introduce them to cancer risk factors associated with HPV, and to inform them on maintaining a healthy lifestyle. We utilized survey data that was collected from an evaluation questionnaire about HPV knowledge from students who attended a Schooling Cancer session at a middle school in Pittsylvania County. The Schooling Cancer Program was geared towards educating middle school students (ages 11–15 years) about various topics, mainly focusing on HPV prevention mechanisms and essential health concepts to live a healthy lifestyle. Inclusion criteria included all students who attended a middle school in Pittsylvania County at the time of this program. No students were excluded from participation in the program activity.

Participants completed a 10-item survey that evaluated student’s general knowledge, cancer risk, and activities that can decrease their lifetime risk of developing cancer. Participants were provided information from the Schooling Cancer Program and asked to complete a post-survey to understand if this information was understood by students. Each correct answer was given one point. For multiple correct answers, the proportion of correct answers selected was awarded in points. Participants could score up to 10 points on this scale. Questions without a response were given a score of 0; however, participants who did not respond to any post-survey questions were considered to be missing data. Demographic information collected included age, gender, Hispanic or Latino origin, and race. Summary statistics are reported in the form of frequencies and proportions for categorical variables. The evaluation questionnaire surveys were collected from the students after the session, and descriptive analyses were conducted using Microsoft Excel.

### 1.3. Ethical issue

The study was approved by the Virginia Commonwealth University Institutional Review Board.

## 2. Results

Since 2017, the CRRCs Outreach team spoke with over 2000 middle school and high school students about a wide range of topics regarding HPV and its link to cancer, as well as how the HPV vaccine reduces the risk of this infection. The evaluation survey asked about students’ age, gender, and race/ethnicity. The survey followed up with a series of questions to assess tobacco knowledge, E-cigarette knowledge, HPV knowledge, and methods to decrease cancer risk.

A total of 169 students responded to the survey questionnaire. Of the students who responded, 87% agreed that tobacco products are associated with various cancers in the body and 81% of students did not agree that E-cigarettes are proven to be safer than cigarettes. 84% of students correctly identified the factors that can decrease their lifetime risk of developing cancer, such as staying physically active, maintaining a healthy weight, and having a balanced diet (Table 1).

HPV knowledge (79.9%) was lower than tobacco knowledge (87.0%), and nearly 80% of these students correctly identify with HPV as the most common STI. Of the students who answered the HPV knowledge question, a higher percentage of females (53.3%) than males (39.3%) correctly stated that HPV is the most common STI. Among Blacks, a larger percentage (28.8% vs 19.4%); while among Whites, a smaller percentage (53.0% vs 56.5%) correctly answered the HPV knowledge question (Table 2).

## 3. Discussion

Cancer prevention education in rural areas is not prioritized, which is why it is important to implement more school-based health education intervention programs. There is growing research that shows that Family Life school-based interventions as well as physician recommendations can help children in rural communities acquire adequate HPV knowledge. Although physicians do not directly communicate with children about getting the HPV vaccine because minors are not able to make these types of medical decisions on their own, children can help inform their parents’ decision on whether their child receives the vaccine. By allowing children to inform their parents on their own HPV knowledge that they learned in school, this may guide their parents to consider the vaccine. Although we did not have pre-evaluation surveys to assess the students’ prior knowledge, the post-evaluation results showed most students who completed a Schooling Cancer session correctly answered the questions about the topics covered. For future studies, having a pre-evaluation knowledge survey on the health-related topic being studied would allow for a more concise comparison of baseline knowledge compared to knowledge learned from the health-related program session.

To improve adolescent knowledge on the HPV vaccine as well as HPV-related diseases in rural communities, school-based intervention needs to take place. To educate local public-school students, we have taken the opportunity to inform students in Pittsylvania County about the risks associated with HPV and its long-term health complications, as well as the benefits of adapting to a healthy lifestyle early on. In Virginia, some schools require certain vaccinations, but not all schools require the HPV vaccine, which is a huge, missed opportunity for children attending schools in Virginia to become vaccinated. By implementing these school-based intervention programs, we have raised awareness about HPV and the vaccine among middle and high school students in Pittsylvania County—ultimately receiving positive feedback.

In addition to school-based intervention, such as Family Life classes, another way to encourage HPV vaccine uptake among adolescents is through physician recommendations. The number one reason that children get vaccinated comes from a physician’s recommendation to the child and their parent, so we have done a substantial amount of work to inform healthcare providers in Southern Virginia about HPV’s link to cancer. By combining school-based interventions with physician recommendations, parents are more informed to vaccinate their children. These two powerful tools are used to increase HPV prevention among adolescents and their primary caregivers. Such methods have allowed us to reach rural communities who do not have as much access to these sources of information, and it is essential that future researchers understand the need to reach out to underserved communities who are unable to receive these resources elsewhere. The Schooling Cancer program has ultimately allowed us to incorporate school-based interventions into the Pittsylvania School systems to raise awareness about HPV prevention and important health concepts to children ages 11–15.

### 3.1. Lessons learned

The aim of this study was to summarize the importance of school-based interventions by educating the youth about the effects of HPV and its related cancers, HPV prevention, and tips for a healthy lifestyle. In rural and underserved communities, we see an ongoing barrier related to healthcare access and adequate healthcare information. HPV vaccination uptake varies upon different geographic regions, with rural regions being medically underserved areas, in comparison to urban areas which have higher rates of vaccine initiation and completion due to healthcare access and knowledge [18]. The Schooling Cancer program has contributed to engaging students in Pittsylvania County on the public health crisis of HPV and its related cancers by utilizing school-based interventions, such as Family Life classes. Through the Schooling Cancer Program study, we have learned that HPV knowledge should be integrated into the school-based Family life courses to increase the acceptability of the HPV vaccine, specifically in rural communities [12]. School-based interventions and physician’s vaccination recommendations were seen to be the most effective ways to increase HPV knowledge among students and their guardians. There are missed opportunities to provide tailored school-based programs to meet the needs of students who may be younger (ages 11–12) and need additional clarification than older students (ages 13–15).

## 4. Conclusions

Through the Schooling Cancer initiative, middle school students in Pittsylvania County learned about essential health concepts and cancer risk factors, including HPV. Without pre-evaluation surveys, we do not know the increase in knowledge, but the post-evaluation results show most students who’ve completed the Schooling Cancer session had correctly answered the questions about the topics covered. Such limitations may result in over-estimating the findings related to the effectiveness of the educational program. Since the study included a convenience sample, we are unable to determine if this program is transferrable to other school settings with similar characteristics. School systems can utilize programs such as Schooling Cancer to educate students on key health topics, such as HPV risk and prevention mechanisms. Future programs should develop ways to effectively engage rural residents and their health care providers about the importance of HPV vaccination in order to improve health equity in cancer.

## Figures and Tables

**Table 1.** Evaluation survey.

	Number of students (%)	
**Age (years)**		
11	22	(13.0)
12	54	(32.0)
13	46	(27.2)
14	34	(20.1)
15	2	(1.2)
Do not know	11	(6.5)
**Sex**		
Male	70	(41.4)
Female	88	(52.1)
Do not know	11	(6.5)
**Race/Ethnicity**		
African American/Black	46	(27.2)
White/Caucasian	91	(53.8)
Hispanic/Latino	7	(4.1)
Asian/Pacific Islander	2	(1.2)
Other	23	(13.6
**True/False Multiple-Choice Question**		
**Tobacco Knowledge (Tobacco products are associated with which of the following)**		
Lung Cancer	17	(10.1)
Heart Disease	0	(0.0)
Oral Cancer	2	(1.2)
Cancer in organs throughout the body	3	(1.8)
All of the above	147	(87.0)
**E-Cigarette Knowledge (E-cigarettes, vapes, and JUULs are scientifically proven to be safer than cigarettes)**		
True	17	(10.1)
False	137	(81.1)
Not Sure	15	(8.9)
**HPV Knowledge (HPV is linked to which of the following cancers)**		
True	135	(79.9)
False	11	(6.5)
Not Sure	23	(13.6)
**Risks of Developing Cancer (Which of the following can decrease your lifetime risk of developing cancer)**		
Staying physically active	5	(3.0)
Staying at a healthy weight	1	(0.6)
Eating a healthy diet, e.g., more fiber	10	(5.9)
Avoiding tobacco products	11	(6.5)
All of the above	142	(84.0)

**Table 2.** HPV knowledge question survey.

HPV is the most common STI		
	True (%)	False (%)
Age (years)		
11	11.1	21.1
12	28.9	45.6
13	30.4	10.5
14	21.5	17.5
15	1.4	0
Unknown	6.7	5.3
Sex		
Male	39.3	52.6
Female	53.3	43.9
Do not know	7.4	3.5
Race/Ethnicity		
African American/Black	28.8	19.4
White/Caucasian	53.0	56.5
Hispanic/Latino	5.3	0
Asian/Pacific Islander	1.5	0
Other	11.4	24.2
